# Mobile App Use for Insomnia Self-Management in Urban Community-Dwelling Older Korean Adults: Retrospective Intervention Study

**DOI:** 10.2196/17755

**Published:** 2020-08-24

**Authors:** Kyungmi Chung, Seoyoung Kim, Eun Lee, Jin Young Park

**Affiliations:** 1 Department of Psychiatry, Yonsei University College of Medicine Yongin Severance Hospital Yonsei University Health System Yongin Republic of Korea; 2 Center for Digital Health Yongin Severance Hospital Yonsei University Health System Yongin Republic of Korea; 3 Institute of Behavioral Science in Medicine, Yonsei University College of Medicine Yonsei University Health System Seoul Republic of Korea; 4 Nowon Eulji Medical Center Eulji University Seoul Republic of Korea; 5 Department of Psychiatry, Yonsei University College of Medicine Severance Hospital Yonsei University Health System Seoul Republic of Korea

**Keywords:** sleep hygiene, cognitive behavioral therapy, sleep initiation and maintenance disorders, telemedicine, mobile apps, treatment adherence and compliance, health education, health services for the aged, community mental health services, health care quality, access, and evaluation

## Abstract

**Background:**

As an evidence-based psychotherapy for treating insomnia, cognitive behavioral therapy for insomnia (CBT-I), which helps people with sleep problems to change their unhelpful sleep-related beliefs and habits, has been well-established in older adults. Recently, the utilization of mobile CBT-I apps has been getting attention from mental health professionals and researchers; however, whether mobile CBT-I apps are usable among older users has yet to be determined.

**Objective:**

The aims of this study were to explore the relationships between subjective sleep quality and subjective memory complaints and depressive symptoms; to explore the relationship between perceived difficulty in mobile app use and usability of the mobile phone–based self-help CBT-I app, named MIND MORE, in urban community-dwelling Korean older adults; to compare changes in subjective sleep quality from pre-intervention to post-intervention, during which they used the mobile app over a 1-week intervention period; and evaluate adherence to the app.

**Methods:**

During the 2-hour training program delivered on 1 day titled “Overcoming insomnia without medication: How to use the ‘MIND MORE’ mobile app for systematic self-management of insomnia” (pre-intervention), 41 attendants were asked to gain hands-on experience with the app facilitated by therapists and volunteer workers. They were then asked to complete questionnaires on sociodemographic characteristics, subjective evaluation of mental health status (ie, depression, memory loss and impairment, and sleep problems), and app usability. For the 1-week home-based self-help CBT-I using the app (post-intervention), 9 of the 41 program attendants, who had already signed up for the pre-intervention, were guided to complete the given questionnaires on subjective evaluation of sleep quality after the 1-week intervention, specifically 8 days after the training program ended.

**Results:**

Due to missing data, 40 of 41 attendants were included in the data analysis. The main findings of this study were as follows. First, poor subjective sleep quality was associated with higher ratings of depressive symptoms (40/40; ρ=.60, *P*<.001) and memory complaints (40/40; ρ=.46, *P*=.003) at baseline. Second, significant improvements in subjective sleep quality from pre-intervention to post-intervention were observed in the older adults who used the MIND MORE app only for the 1-week intervention period (9/9; *t*_8_=3.74, *P*=.006). Third, apart from the program attendants who did not have a smartphone (2/40) or withdrew from their MIND MORE membership (3/40), those who attended the 1-day sleep education program adhered to the app from at least 2 weeks (13/35, 37%) to 8 weeks (2/35, 6%) without any further contact.

**Conclusions:**

This study provides empirical evidence that the newly developed MIND MORE app not only is usable among older users but also could improve subjective sleep quality after a 1-week self-help intervention period.

## Introduction

### Background

Insomnia and sleep disturbance symptoms negatively influence mental health-related quality of life and functional abilities in older adults, with the perception of nonrestorative and poor-quality sleep [1]. According to the finding of a systematic review and meta-analysis of population-based prospective cohort studies [2], insomnia increases the risk of incident all-cause dementia in elderly individuals. Poor sleep quality may result in increased mortality and psychiatric comorbidities in both nondemented and demented elderly, causing stress for their caregivers [3]. Greater sleep disturbance has been correlated with more severe impairments in cognitive function in two cohort groups, particularly patients with Alzheimer disease and even healthy, nondemented elderly; furthermore, both groups with sleep problems experienced more depressive symptoms [4]. Persistent insomnia also appears to be a risk factor for onset of depression in the elderly [5]. With the rapid growth of the aging population in South Korea, sleep problems have increasingly been recognized as an important public health concern among community-dwelling older Korean adults. In particular, the prevalence of insomnia is estimated to be at least 32% in the elderly Korean population aged ≥60 years, with a significantly higher prevalence in women than in men [6,7]. The prevalence rates of insomnia in patients with subjective memory impairment, mild cognitive impairment, and dementia were 23.2%, 19.6%, and 31.0%, respectively, in a community sample of elderly Korean individuals aged ≥65 years [8]. Additionally, more than 50% of patients with insomnia report depressive symptoms [7]. Given the high prevalence of late-life insomnia worldwide, there is a need to better understand the associations between sleep quality and individual psychiatric comorbidities such as cognitive dysfunction and depression.

As approximately one-third of Korean older adults experience insomnia [7], its treatment is of great importance for both patients with insomnia and those who are concerned about their sleep-related health behaviors — to effectively manage sleep problems and improve sleep quality as well as prevent or reduce late-life disability [9]. It is important to consider long-term outcomes of any treatments for late-life insomnia even after treatment discontinuation as well as short-term outcomes during treatment administration because insomnia is often recurrent or persistent [10]. A number of previous studies have revealed that cognitive behavioral therapy for insomnia (CBT-I) alone was more effective than pharmacotherapy alone or combined treatment after treatment administration and discontinuation [10-15]. Although pharmacotherapy is well-known as the most frequently recommended intervention for insomnia, moderate efficacy has been shown while sleep medications were being used, and changes in sleep quality returned to baseline after discontinuing the medications [12,16]. In addition, the long-term use of sedative-hypnotics is contraindicated due to the diverse and unwanted adverse effects such as falls, nausea, confusion, dizziness, headache, daytime drowsiness, abuse and dependence, memory impairment, or rebound insomnia [12,13,17]. To deal with the potential adverse effects of sleep medicines, short-term, intermittent use or an alternative intervention based on CBT-I is recommended in older adults. Considering that psychological approaches produce sustained outcomes without the risk for tolerance or side effects related to pharmacologic approaches [13,15], CBT-I can be regarded as a more appropriate, evidence-based, first-line treatment option than pharmacotherapy, with clinically meaningful effect sizes. Defined as a multimodal therapy delivered in person, a combined CBT-I intervention, which incorporates at least 2 of the 5 widely accepted cognitive (cognitive strategies), behavioral (stimulus control, sleep restriction, relaxation), and educational (sleep hygiene education) components, is more preferred than a standalone component [15,18-20].

CBT-I results in significant improvements in sleep onset latency, wake after sleep onset, total sleep time, and sleep efficiency [15]; however, its efficacy might be reduced by such adherence issues as drop-out, premature termination, irregular attendance to sessions, and noncompliance to homework [21]. As mobile health (mHealth) technology has recently become more accessible, elderly adults are more familiar and comfortable with mobile devices in their everyday lives and natural settings [22]. Since adherence to a CBT-I protocol leads to better treatment outcomes [23], a promising, readily accessible approach to address these adherence issues can be the adoption of a mobile app–delivered CBT-I for the self-management of insomnia among older adults. Based on the stepped care model of health care delivery with two fundamental features [24,25], the pure self-help or guided self-help CBT-I app and its dashboard could serve as the “least restrictive” therapy with evidence-based, entry-level treatment to achieve significant health gains using a minimal intervention principle at the lowest cost and with the lowest required treatment intensity (ie, the least specialist therapist time) and as a “self-correcting” mechanism to systemically monitor and measure treatment progress and outcome, respectively. Despite the growing interest in using mobile phones and services among the elderly, individual attributes, such as gender, education, technology self-efficacy and anxiety, and age-related health and ability characteristics, have direct effects on technology acceptance behavior [26]. In addition, 3 distinct factors, namely self-efficacy, conversion readiness, and peer support, affect older adults’ technology acceptance behaviors, particularly the “intention to learn” phase [27]. Senior users tend to use their mobile phones for very limited purposes and discourage themselves from learning new technology through trial and error, thereby encountering more learning difficulties [28]. To enhance adherence to a newly developed CBT-I app installed on their own mobile phones, it is important to help senior users overcome the fear of learning a new mHealth technology and to provide them with more appealing features and useful, easy-to-use functionality.

Previous studies have focused on assessing the efficacy, feasibility, and usability of CBT-I apps to complement standard treatment for insomnia, such as sleep medication use or traditional CTB-I programs [29-32]; they have not focused on how to lower the barriers to the acceptance of a mobile app–based CBT-I by older adults, who often have learning difficulties in the initial phase. In terms of age-related barriers to usability evaluations with older adults, half of the identified high-level usability issues with mHealth app use were related to motivational barriers, such as low computer literacy and low trust in their own ability to use the apps, more than cognitive and perceptional barriers, such as working memory and visual acuity [33]. Particularly, when it comes to using digital technology to support mental health, the following may negatively affect the readiness of older adults to engage with technology: low mood, fear of consequences, perceived superiority of human contact, and a lack of prior knowledge, skills, or experience applicable to mobile devices and mHealth app usage [34]. Based on findings from relevant studies, motivational barriers should receive more attention in usability research in the field of mobile public health for older people. As there has been a high uptake of educational programs in community centers (eg, Community Mental Health Welfare Center and Seoul Metropolitan Center for Dementia) to improve quality of life in older community dwellers and to promote geriatric mental health for the prevention or early detection of a transition from nonclinical to clinical psychotic states [35], utilizing this public health infrastructure can allow older adults to easily access and efficiently learn and adhere to new CBI-I apps without active, constant intervention from health professionals.

### Objectives

To examine the treatment effects of a mobile insomnia self-management intervention, this retrospective study analyzed data sourced from an urban community center for dementia prevention in South Korea. In the center, a mobile phone–based CBT-I app (hereafter “MIND MORE”), which was designed and developed by our research team, was used during a 1-day sleep education program. The primary purpose of this study was to determine whether self-management of insomnia is facilitated with the MIND MORE mobile app over a 1-week intervention period by comparing changes in subjective sleep quality from pre-intervention to post-intervention, particularly in community-dwelling Korean older adults. We hypothesized that there would be improvements in subjective sleep quality from pre-intervention to post-intervention. Secondary aims of this study were to evaluate adherence to the MIND MORE app, explore the relationship between perceived difficulty in mobile app use and usability of the MIND MORE app, and explore the relationships between subjective sleep quality and subjective memory complaints and depressive symptoms.

## Methods

### Participant Recruitment

A total of 41 older adults, all of whom registered at the Seoul Metropolitan Center for Dementia (SMCD) located in Seodaemun-gu, Seoul, South Korea, signed up for a 2-hour 1-day training program titled “Overcoming insomnia without medication: How to use the ‘MIND MORE’ mobile app for systematic self-management of insomnia” (pre-intervention). The training program was held on August 8, 2019 in an auditorium at the SMCD, which aims to provide preventative solutions for geriatric residents at normal or high risk of developing geriatric mental illness (not only dementia) as well as early detection for better treatment, rehabilitation support, and appropriate management in those at different stages of illness. For the 1-week home-based self-help CBT-I using the MIND MORE app (post-intervention), a therapist recruited a limited number of 9 volunteers from the entire 41 attendants of the 1-day training program. As the program was not provided for the purpose of academic research, program attendants gave their written consent for their participation, and those who lacked the capacity to provide their own consent to participate in the program were required to provide surrogate consent through the person who was a legally-authorized representative, particularly under the supervision of the SMCD.

To sign up for the center’s program, the following requirements had to be satisfied: (1) male and female senior residents in Seodaemun-gu (aged ≥60 years); (2) either possess a smartphone or a feature phone (with more limited computing capabilities than smartphones) but were interested in smartphone-based self-help treatment for insomnia; and (3) with adequate literacy. For this retrospective study, the study protocol was approved after the program ended by the Institutional Review Board of Severance Hospital, Yonsei University College of Medicine in Seoul, South Korea.

For sensitive health-related information to be protected, the final version of the digitized datasheet (n=41) had personally identifiable information such as name, birthdate, and mobile phone or home number removed and then was forwarded to our research team by the chief therapist who was in charge of the education program. According to the exclusion criteria for the data analysis, 1 of 41 attendants was excluded due to missing data because we had no contact information to follow-up on the missing responses to baseline clinical and other demographic characteristics for that person.

### MIND MORE App

#### Mobile Phone–Based CBT-I App

**Figure 1:**
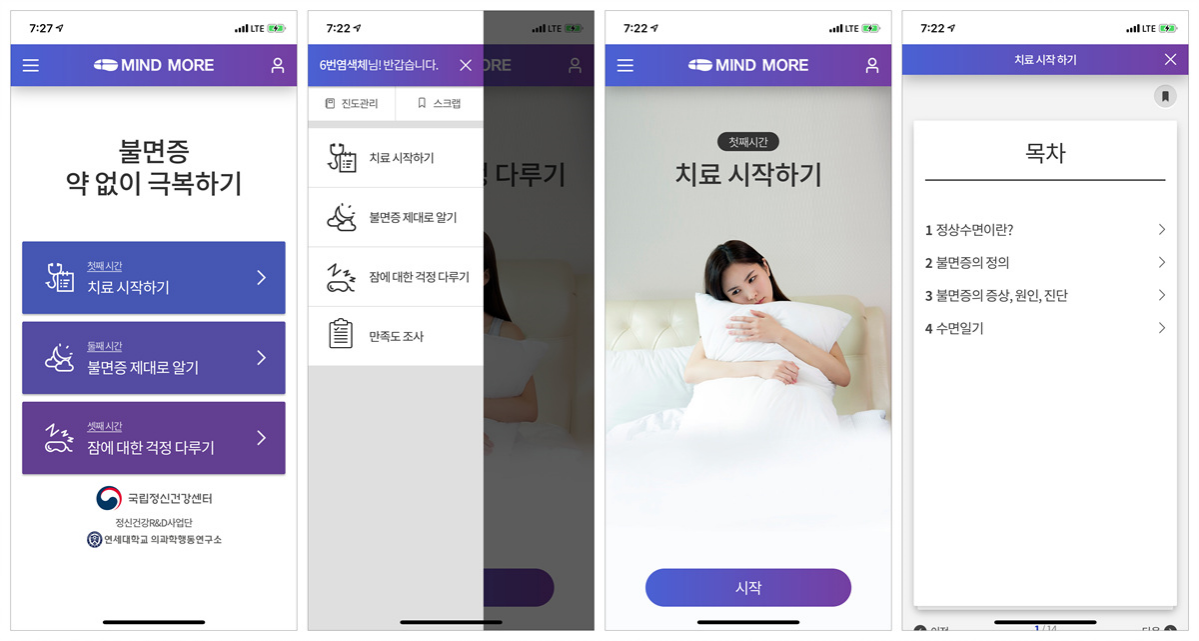
Sleep hygiene education program with 3 main sessions.

**Figure 2:**
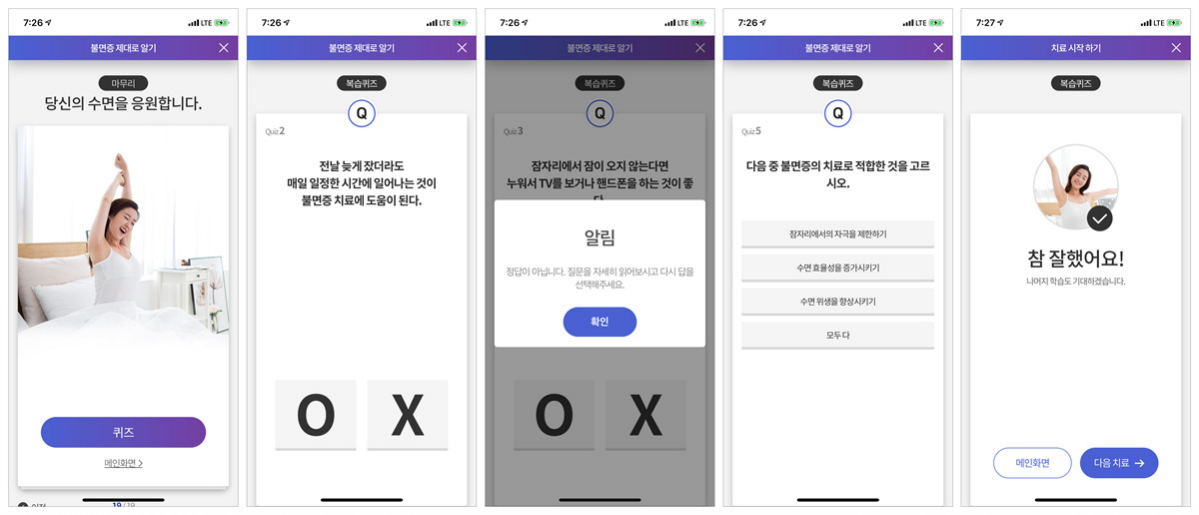
Quiz session after the end of each of the 3 main sleep education sessions.

MIND MORE is a multimodal CBT-I app, with a greater focus on sleep education, with 4 components: (1) sleep hygiene education, (2) sleep restriction, (3) stimulus control, and (4) cognitive therapy. In the MIND MORE app, the main sleep hygiene education program is composed of 3 sessions (Figure 1), quizzing its users at the end of each session to evaluate what they learned (Figure 2). The sleep hygiene education helps to better understand the perpetuating mechanisms that sustain insomnia and to correct unhelpful, inflexible sleep-related beliefs and anxiety, conditioned arousal to the bed and bedroom, and sleep-disruptive habits such as daytime napping and spending excessive time in bed [20], which contributes to adherence to treatment recommendations provided by MIND MORE. All the MIND MORE contents referred to the CBT-I program that is currently implemented in the Yonsei University Health System, particularly based on the CBT-I protocol by Edinger and Carney [20].

**Figure 3:**
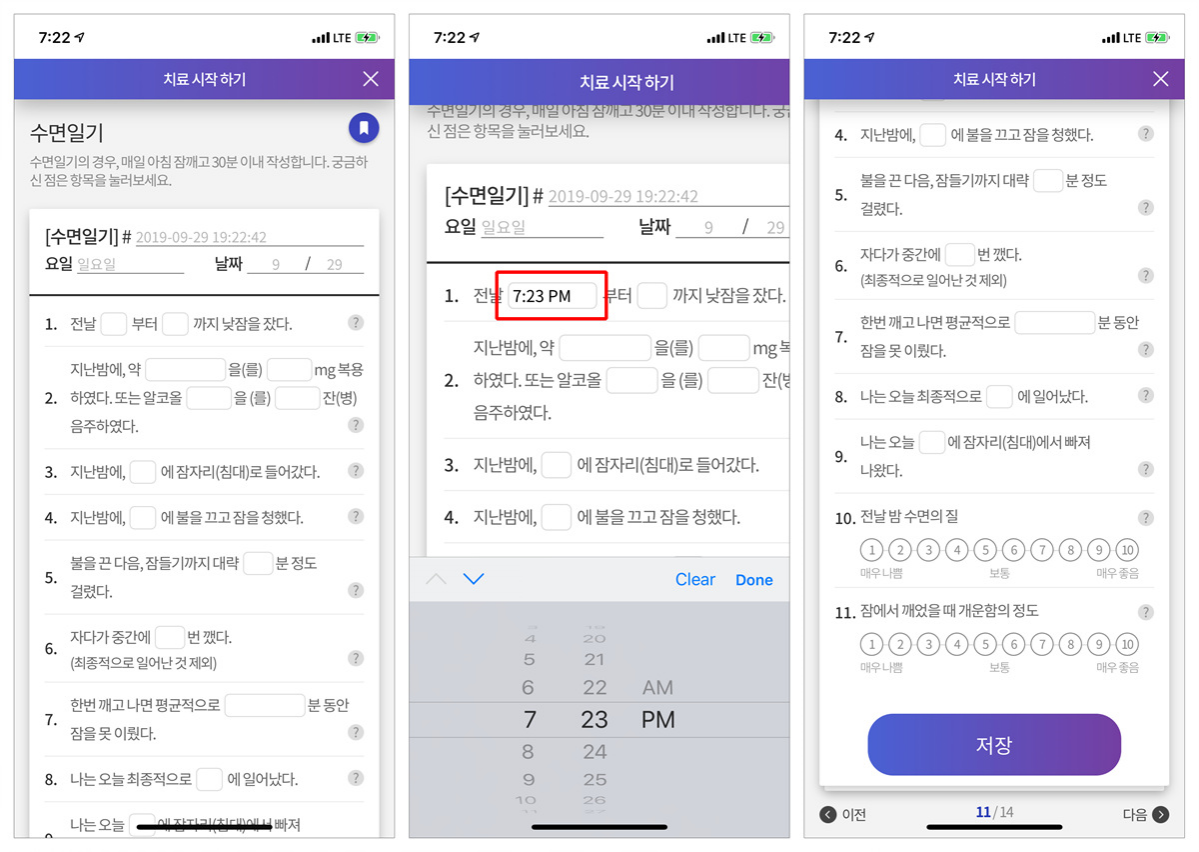
Sleep diary.

**Figure 4:**
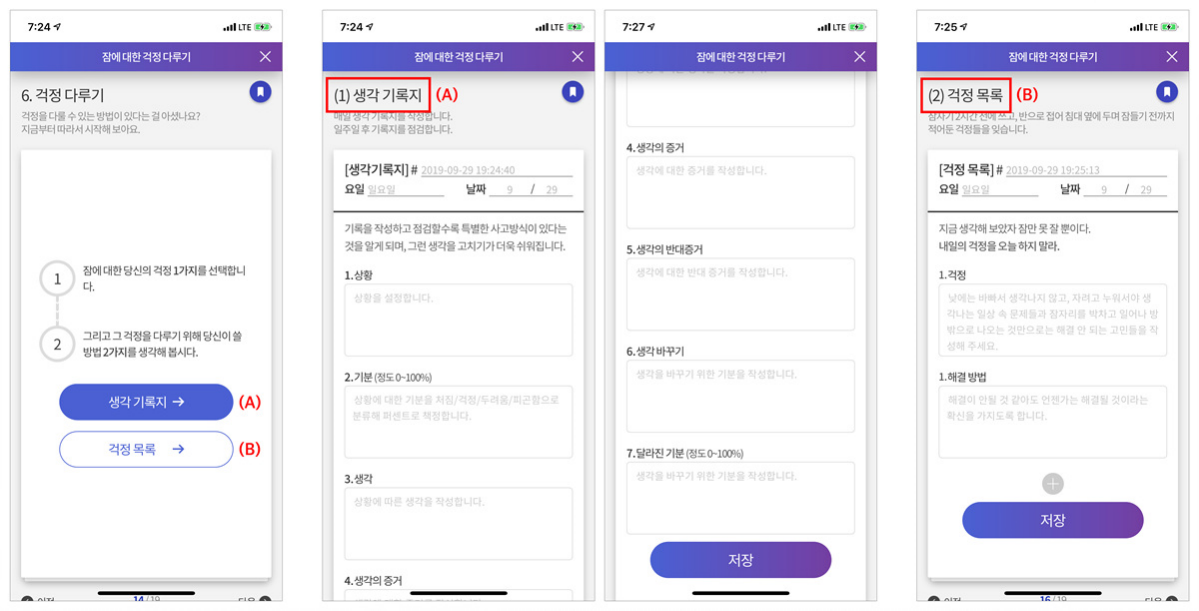
Cognitive therapy with (A) thought record and (B) constructive worry worksheet.

The education was supplemented by a sleep diary and cognitive therapy, as well as sleep restriction and stimulus control contents. As a valuable tool for assessing insomnia, a sleep diary can prospectively monitor sleep habits and patterns over time (Figure 3), thus identifying good candidates for implementing cognitive and behavioral therapy strategies based on the data [20]. More importantly, previous research addressing the issue found that some older adults had difficulty in keeping pencil-and-paper sleep diaries, thereby leading to missing values [36]. It was expected that this issue might be handled by technical support from mobile technology. In addition to keeping a sleep diary, the cognitive therapy included recording a “thought record” for cognitive restructuring and a “constructive worry worksheet” for worry control (Figure 4), helping elderly users to easily complete their sleep information in the formatted blank spaces.

**Figure 5:**
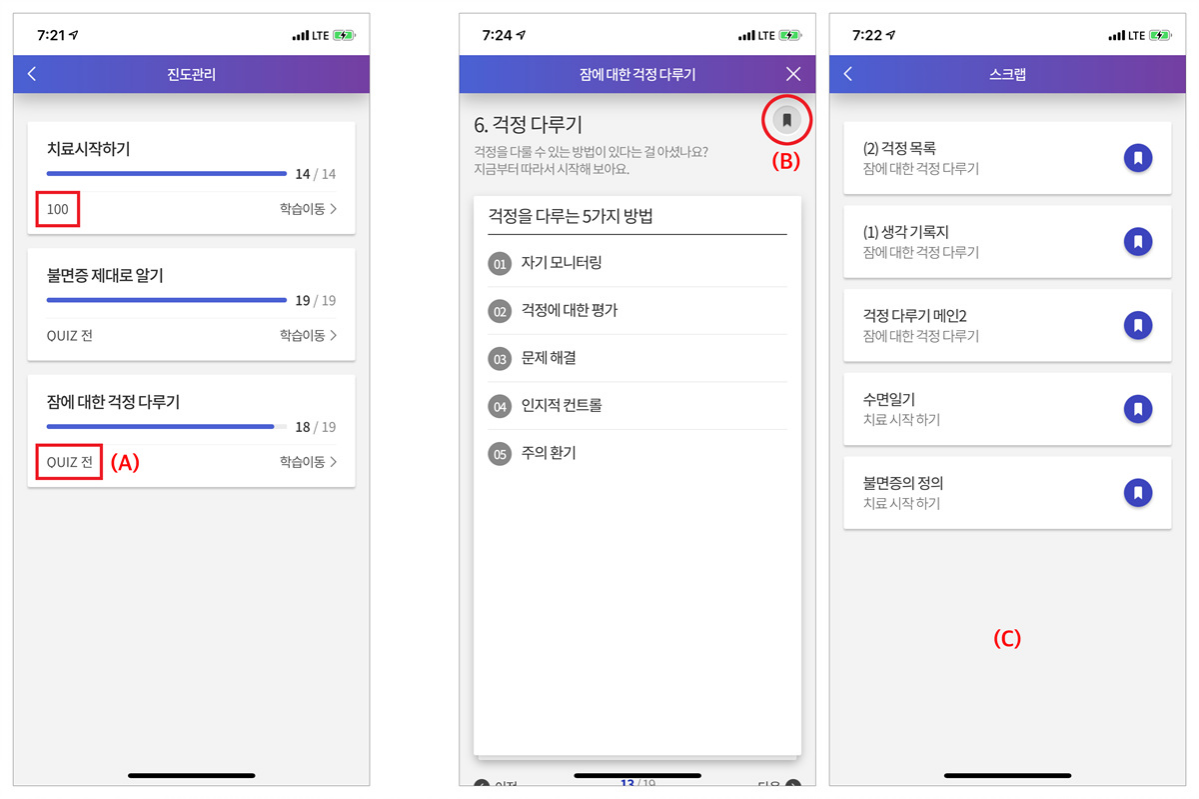
Additional features of the MIND MORE app: (A) learning progress management, (B) clipping button, and (C) list of clipped pages.

To increase the accessibility of information, the MIND MORE app provided its users with additional features such as “learning progress management” to monitor the progress rate and quiz scores from each of the 3 sessions and “clipping” to add specific pages containing important information to their list of “Scrap” and to directly access clipped pages (eg, sleep diary, thought record, or constructive worry worksheet), particularly in the hamburger menu (Figure 5). The MIND MORE app can be downloaded in both the Google Play store for Android phone users and the App Store for Apple phone users. Multimedia Appendices 1–5 provide all MIND MORE app figures translated to English.

#### Dashboard

Based on the stepped care model [24,25], the MIND MORE app was initially designed to only allow users to reflect on the logs of their sleep diary, thought record, and constructive worry worksheet. If there were few changes in sleep quality, the users were expected to share all the logs with therapists or clinicians with whom they had established good rapport and then to receive more direct, systematic management, thus changing a pure self-help intervention into a therapist-guided self-help intervention. In this context, its dashboard was limited to display the following information: (1) registration and last login dates, (2) learning progress checks including last learning dates and quiz scores in each of the 3 sessions, and (3) behavioral and cognitive therapy records including the last dates and number of times users completed their sleep diary, thought record, and constructive worry worksheet as well as the total number of written constructive worries. If the users withdrew from MIND MORE membership, their data were no longer displayed in the dashboard and were removed from the server.

### Procedure

The entire procedure for this retrospective study was as follows. At the SMCD in Seodaemun-gu, a 2-hour training program for use of the MIND MORE CBT-I app was held over the course of 1 day; during the program, the attendants gained hands-on experience with the MIND MORE app with the help of therapists and volunteer workers, from installing and signing up for the app to learning its functions and features (pre-intervention). At the end of the program, all 41 attendants were required to complete the pencil-and-paper questionnaires on sociodemographic characteristics and subjective evaluation of mental health status (ie, depression, memory loss and impairment, and sleep problems) and usability of the MIND MORE. Of the 41 attendants, 9 were enrolled in the 1-week home-based self-help CBT-I where the 9 attendants used the MIND MORE app for a week based on what they learned during the 1-day training program. They were guided to fill out and submit the given pencil-and-paper questionnaires on subjective evaluation of sleep quality after the 1-week program (pre-intervention), specifically 8 days after the 1-day training program ended (post-intervention). For the 8-week follow-up period, adherence of all attendants to the app was monitored in the dashboard without any direct therapist contact. For this retrospective study, our research team was provided with a coded datasheet by the SMCD.

### Subjective Measurements

#### Short-Form Geriatric Depression Scale

The 15-question brief version of the Geriatric Depression Scale, known as GDS-15, is a widely used screening measure for depression in the geriatric population [37,38]. In this study, the Korean version of the GDS-15 (SGDS-K) was employed in the community-dwelling elderly in Korea, with an optimal cut-off point of 8 for both normal and clinical populations [39,40]. All attendants were asked to answer each question of the 15-item SGDS-K with either “yes” (1) or “no” (0) in reference to how they felt over the past week.

#### Subjective Memory Complaints Questionnaire

The Subjective Memory Complaints Questionnaire (SMCQ) is a brief, reliable, and valid questionnaire for the global assessment of memory function (global memory) and the specific judgment of memories of particular events (everyday memory), with the ability to screen for dementia [41]. The SMCQ consists of a total of 14 items: 4 items for global memory and 10 items for everyday memory. Each item is answered with either “yes” (1) or “no” (0), and the optimal cut-off point for dementia is 5 for elderly without dementia and 6 for demented elderly.

#### Pittsburgh Sleep Quality Index

The Pittsburgh Sleep Quality Index (PSQI) is composed of 19 self-rated questions and 5 questions rated by a roommate or bed partner (if possible), and only self-rated questions are used for the scoring [42]. According to the given scoring sheet, the 19 self-rated items are combined to calculate the following 7 components: (1) subjective sleep quality, (2) sleep latency, (3) sleep duration, (4) habitual sleep efficiency, (5) sleep disturbance, (6) use of sleeping medication, and (7) daytime dysfunction. Each component score is coded as 0, 1, 2, or 3. Then, the 7 component scores are summated to yield the global PSQI score. This study administered the Korean version of the PSQI (PSQI-K) using the best cutoff score of 8.5 [43]. All questions were related to usual sleep habits for the past month only, and attendants were guided to choose the appropriate reply for the majority of days and nights during the past month as accurately as possible.

#### Perceived Difficulty in Mobile App Use

To evaluate perceived difficulty in mobile app use, all elderly attendants both with and without their own smartphones responded to the question of “Do you have difficulty in installing, updating, and removing mobile apps on your own smartphone by yourself?” scored on a 5-point scale anchored by 0 (not at all), with scores ranging to 4 (always). In particular, feature phone users were guided to imagine that they would have their own smartphone and then answer the question. This question was included in the questionnaire for demographic information.

#### Usefulness, Satisfaction, and Ease of Use Questionnaire

To assess software usability, we used the Usefulness, Satisfaction, and Ease of Use (USE) questionnaire [44], consisting of 4 subfactors: usefulness (5 items), ease of use (4 items), ease of learning (3 items), and satisfaction (5 items). All attendants were asked to rate a series of attitude statements for agreement, using 5-point Likert rating scales ranging from 0 (strongly disagree) to 4 (strongly agree). The arithmetic mean was used to calculate each subfactor score and the total usability score.

### Statistical Analysis

All statistical analyses were performed using PASW Statistics 18.0 software (SPSS Inc. Chicago, IL). To test the normal distribution of variables, *Z* scores for skewness (ie, skewness/SE of skewness) and kurtosis (ie, kurtosis/SE of kurtosis) were calculated. If the sample size is small (N<200), an absolute *Z* score value >1.96 is insufficient to assume normality of the data (*P*<.05), which means that a nonparametric statistical test should be used, based on the rule of thumb for normality testing by Ghasemi and Zahediasl [45]. According to the results of the normality tests for the 40 1-day program attendants, the normality assumptions of the SGDS-K, SMCQ, and PSQI-K scores were violated, while all the scores for the perceived difficulty in mobile app use and the USE questionnaire including the 4 subfactors and total usability scores met the assumption. For the 9 attendants in the 1-week home-based self-help CBT-I using the MIND MORE app, the PSQI-K scores for both pre-intervention and post-intervention were normally distributed. Hence, the correlation between variables was determined using statistical tests: the Spearman rank correlation as a nonparametric statistical test and Pearson correlation as a parametric statistical test. The paired-samples *t* test, as a parametric method, was used for hypothesis testing.

## Results

### Participant Characteristics

Due to the occurrence of missing data, 40 (35 women) of 41 program attendants, aged 64 to 86 years (mean 75.75 years, SD 5.87 years), were included in the data analysis. Of the 40 attendants, the mean age of the 9 female attendants who were involved in the 1-week home-based self-help CBT-I using the MIND MORE app was 71.56 years (SD 4.36 years; range 65-78 years). This study sample was highly educated in that the majority of the attendants (25/40, 63%) reported that the highest level of education was high school. In terms of the prevalence of depressive symptoms, memory complaints, and sleep problems at baseline, 3 (3/40, 8%), 7 (7/40, 18%), and 15 (15/40, 38%) had scores greater than the cut-off points for the SGDS-K, SMCQ, and PSQI-K, respectively.

In terms of mobile phone possession, 38 had Android smartphones (38/40, 95%) including the brands Samsung (29/38, 76%) and LG (9/38, 64%), and the rest had feature phones (2/40, 5%) on which mobile apps were not available. Regardless of the type of phone the elderly attendants owned, the amount of time they spent using their own mobile phones was reported as follows: <1 hour (20/40, 50%; 2 feature phone users were included), 1-2 hours (12/40, 30%), 2-3 hours (2/40, 5%), 3-4 hours (3/40, 8%), and >4 hours (3/40, 8%). In addition to these basic demographic characteristics, more detailed information is presented in Table 1.

**Table 1:** Demographic characteristics of the study sample.

Characteristics		One-day training program for MIND MORE use (n=40)	One-week self-help intervention with MIND MORE (n=9)
**Gender, n (%)**			
	Male	5 (13)	0 (0)
	Female	35 (88)	9 (100)
**Age, n (%)**			
	Young-old (60-69 years)	8 (20)	4 (44)
	Old-old (70-79 years)	22 (55)	5 (56)
	Oldest-old (80-89 years)	10 (25)	0 (0)
**Marital status, n (%)**			
	Married	23 (58)	3 (33)
	Widowed	17 (43)	6 (67)
**Occupation, n (%)**			
	Employed (full-time)	2 (5)	2 (22)
	Employed (part-time)	1 (3)	0 (0)
	Housewife	25 (63)	6 (67)
	Retired	3 (8)	0 (0)
	Unemployed	9 (23)	1 (11)
**Educational level, n (%)**			
	Elementary school	6 (15)	1 (11)
	Middle school	9 (23)	3 (33)
	High school	14 (35)	5 (56)
	College (2-3 years)	3 (8)	0 (0)
	University (4-5 years)	6 (15)	0 (0)
	Postgraduate (Masters/Doctoral)	2 (5)	0 (0)
**Psychiatric diagnosis (yes)^a^, n (%)**			
	Alzheimer’s disease	3 (8)	1 (11)
	Major depressive disorder	5 (13)	0 (0)
	Anxiety disorder	1 (3)	0 (0)
	Sleep disorder	1 (4)	0 (0)
SGDS-K^b^ (score), mean (SD)		2.00 (2.73)	1.00 (1.12)
SMCQ^c^ (score), mean (SD)		2.33 (2.67)	1.67 (1.58)
PSQI-K^d^ (score), mean (SD)		7.50 (4.12)	8.00 (2.50)
Perceived difficulty in mobile app use (score), mean (SD)		2.53 (1.28)	2.11 (1.17)
**Social support for mobile phone use (yes)^a^, n (%)**			
	Child	25 (63)	8 (2)
	Grandchild	8 (20)	2 (5)
	Daughter-in-law	3 (8)	1 (3)
	Spouse	3 (8)	0 (0)
	Acquaintance	7 (18)	2 (5)
	None	2 (5)	0 (0)

^a^Multiple responses were allowed.

^b^SGDS-K: Korean version of the 15-question Geriatric Depression Scale (cut-off score of 8).

^c^SMCQ: Subjective Memory Complaints Questionnaire (cut-off score of 5 for elderly without dementia and 6 for demented elderly).

^d^PSQI-K: Korean version of the Pittsburgh Sleep Quality Index (cut-off score of 8.5).

### Relationships Between Subjective Sleep Quality and Subjective Memory Complaints and Self-Reported Depressive Symptoms

**Figure 6:**
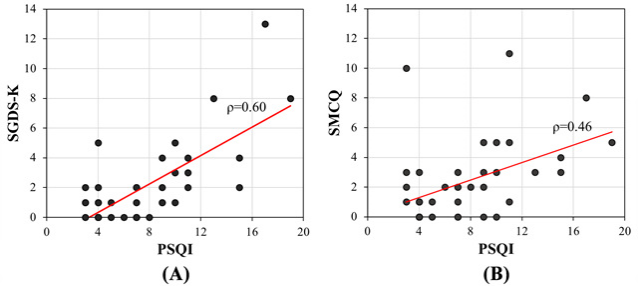
Scatter plots showing the Spearman rank correlations (n=40) between (A) subjective sleep quality and subjective memory complaints and (B) subjective sleep quality and self-reported depressive symptom severity. PSQI-K: Korean version of the Pittsburgh Sleep Quality Index; SGDS-K: Korean version of the 15-question Geriatric Depression Scale; SMCQ: Subjective Memory Complaints Questionnaire.

As shown in Figure 6, the Spearman rank correlation coefficients between the PSQI-K and SGDS-K scores and between the PSQI-K and SMCQ scores were .60 (*P*<.001) and .46 (*P*=.003; 2-tailed), respectively, for all 40 elderly attendants. Both showed moderate positive correlations; however, subjective sleep quality of individuals was more strongly associated with change in affective state than in cognitive function.

### Relationship Between Perceived Difficulty in Mobile App Use and Usability of MIND MORE

Table 2 shows the scores for perceived difficulty in using the mobile app and usability (with the 4 subfactors: usefulness, ease of use, ease of learning, and satisfaction) of the MIND MORE app, and Table 3 shows the results of the Pearson correlation analysis (2-tailed) between the scores for perceived difficulty in using the mobile app and usability (with the 4 subfactors: usefulness, ease of use, ease of learning, and satisfaction). There was only the significant negative correlation between perceived difficulty and ease of learning; ease of learning was significantly, positively correlated with ease of use, satisfaction, and usability scores.

**Table 2:** Perceived difficulty in using the mobile app and Usefulness, Satisfaction, and Ease of Use (USE) questionnaire score and subfactor scores (n=40).

Factor	Scores, mean (SD)
PD^a^	2.53 (1.28)
Usefulness	2.90 (0.51)
EOU^b^	2.01 (0.86)
EOL^c^	1.85 (0.72)
SATIS^d^	2.56 (0.68)
Total USE score	2.33 (0.56)

^a^PD: perceived difficulty in using the mobile app.

^b^EOU: ease of use.

^c^EOL: ease of learning.

^d^SATIS: satisfaction.

**Table 3:** Pearson correlation coefficients of the relationships between perceived difficulty in using the mobile app and the Usefulness, Satisfaction, and Ease of Use (USE) questionnaire score and subfactor scores (n=40).

Factor		PD^a^	Usefulness	EOU^b^	EOL^c^	SATIS^d^	Total USE score
**PD**							
	*r*	1	0.13	–0.17	–0.37	0.13	–0.12
	*P* value	—^e^	.42	.28	.02	.44	.46
**Usefulness**							
	*r*	0.13	1	0.32	0.29	0.50	0.60
	*P* value	.42	—^e^	.047	.07	<.001	<.001
**EOU**							
	*r*	–0.17	0.32	1	0.79	0.62	0.91
	*P* value	.28	.047	—^e^	<.001	<.001	<.001
**EOL**							
	*r*	–0.37	0.29	0.79	1	0.39	0.82
	*P* value	.02	.07	<.001	—^e^	.013	<.001
**SATIS**							
	*r*	0.13	0.50	0.62	0.39	1	0.79
	*P* value	.44	<.001	<.001	.013	—^e^	<.001
**Total USE score**							
	*r*	–0.12	0.60	0.91	0.82	0.79	1
	*P* value	.46	<.001	<.001	<.001	<.001	—^e^

^a^PD: perceived difficulty in using the mobile app.

^b^EOU: ease of use.

^c^EOL: ease of learning.

^d^SATIS: satisfaction.

^e^Not applicable.

### Comparison of Changes in Subjective Sleep Quality From Pre-Intervention to Post-Intervention

The result of a 2-tailed paired-samples *t* test revealed that there was a significant mean difference in the subjective evaluation of sleep quality between pre-intervention (mean 8.00, SD 2.50) and post-intervention (mean 5.11, SD 1.36), indicating that using the MIND MORE app for a week led to improved sleep quality in the elderly attendants (*t*_8_=3.74, *P*=.006).

### Adherence to the MIND MORE Mobile App

To quantify the adherence rate to the self-help MIND MORE CBT-I app during an 8-week follow-up, the registration and last login dates on the app for all 35 program attendants with their own smartphones were downloaded from the app dashboard. The total number of days from the registration date to the last login date within the 8-week period was counted. As listed in Table 4, total days of use were categorized into 6 groups: <1 day, 1-6 days (1 week), 7-13 days (1-2 weeks), 14-27 days (2-4 weeks), 28-55 days (4-8 weeks), and >56 days (>8 weeks). After that, the adherence rate was determined. In the same way, the adherence rate within the same period for the 9 attendants involved in the 1-week intervention was determined. In Table 4, the results of adherence to the app are presented more in detail.

**Table 4:** Elderly users’ adherence to the MIND MORE app without any direct therapist contact.

Total days of use	One-day training program for MIND MORE use (n=35)^a^, n (%)	One-week self-help intervention with MIND MORE (n=9), n (%)
<1 day (on-site experience)	18 (51)	3 (33)
1-6 days (<1 week)	2 (6)	2 (22)
7-13 days (1-2 weeks)	2 (6)	1 (11)
14-27 days (2-4 weeks)	5 (14)	2 (22)
28-55 days (4-8 weeks)	6 (17)	1 (11)
>56 days (>8 weeks)	2 (6)	0 (0)

^a^Of the 40 attendants, 2 were feature phone users, and 3 withdrew from MIND MORE app service membership; these 5 attendants were excluded.

## Discussion

### Principal Findings

The main findings of this study were as follows. First, the newly developed MIND MORE app was usable among community-dwelling older Korean adults. Second, as hypothesized, subjective sleep quality significantly improved from pre-intervention to post-intervention, particularly by using the mobile phone–based self-help CBT-I app we developed during only a 1-week intervention period. Third, except for the attendants who did not have their own smartphones (2/40) and withdrew from their MIND MORE membership (3/40), those in the 1-day sleep education program adhered to the MIND MORE app for at least 2 weeks (13/35, 37%) to 8 weeks (2/35, 6%) without any further contact.

In line with previous studies mentioned in the Introduction [7,8], this study showed that subjective sleep quality is more closely linked to depressive symptoms than to memory complaints. Given the high prevalence of depression in elderly women [6,7], it is important to help with timely prevention or management of sleep problems. Based on the characteristics of this study sample, most program attendants (35/40, 88%) were women, which is considered a positive outcome given that they are vulnerable to depression and sleep disturbance and are trying to self-manage their mental issues. According to the policy report of the 2017 National Survey of Older Koreans by the Korea Institute for Health and Society Affairs [46], female older adults, who are more likely to have ≥3 comorbidities or be in a poorer health state, were the main users of senior citizen centers and senior welfare centers, compared with male counterparts. In contrast, male older adults were more satisfied with the status of their subjective health, as well as that of their social, leisure, and cultural activities, than female counterparts. In line with these survey findings, this study also revealed that elderly men had a relatively low participation rate in the community center programs, whereas elderly women tried to stay active and seek adequate treatment for their mental health concerns. In this regard, the issue of how to increase accessibility to the MIND MORE app for older male adults would be of great importance to be addressed in future studies.

To determine whether cognitive and behavioral changes were prompted through the mobile app–based CBT-I, the 9 attendants in the 1-week home-based self-help CBT-I intervention were asked to report their user experience with the MIND MORE app by using the given open-ended questionnaires, particularly on the effectiveness of mobile-based sleep hygiene education and the sleep diary. According to the following responses, sleep restriction and stimulus control were used to improve sleep quality (Textbox 1).

Moreover, the sleep diary played a key role in identifying appropriate candidates to implement CBT-I strategies based on the users’ records of sleep habits and patterns over time [20] (Textbox 2).

**Responses regarding sleep restriction and stimulus control to improve sleep quality.**

Since I haven’t drunk coffee late in the evening, I could sleep well.

A4

I only drank a cup of coffee in the morning, and I didn’t take a nap at all.

A6

When I was too tired, I used to take a nap. But…since I tried not to take too much naps, I got to fall asleep well at night.

A3

I was so hard to wake up too early in the morning, but sleep restriction improved my sleep a bit.

A3

**Responses regarding the usefulness of the sleep diary.**

I tried to put what I’ve learned from the program into practice, so my insomnia almost went away these days.

A6

It doesn’t seem to help very much. I don’t take a nap and drink coffee at all, because I'm afraid that I won’t be able to fall asleep.

A7

As reflected in the comments of our respondents, individual differences in sleep problems should be carefully considered when implementing a mobile app–based self-help CBT-I without therapist intervention. To enhance the treatment effects, it is recommended to include a pretreatment assessment session to identify what causes or maintains sleep problems by conducting a clinical interview or completing a sleep history questionnaire or sleep diary before the start of the first session of sleep hygiene psychoeducation. After that, appropriate CBT-I components should be target outcomes, such as automatic thoughts or behaviors.

Most of all, because older adults perceive mobile app use as more difficult in everyday life, they were more likely to have difficulty learning how to use the MIND MORE app; therefore, it would be important to implement more education programs to familiarize elderly adults with mobile devices and apps in community centers, which in turn may positively influence perceived usability and mHealth technology self-efficacy. Consistent with the finding of the study by Lund [44], this study revealed that ease of learning was highly correlated with ease of use. Furthermore, once the elderly novice users perceived that they could easily and quickly learn to use the MIND MORE app (ease of learning), the MIND MORE app was more likely to be perceived as easy to use, as providing satisfaction, and as usable to achieve goals with effectiveness, efficiency, and satisfaction in a specified context to manage sleep problems (usability).

Although it was expected that the app’s high learnability and mobility might contribute to easy access to this digital therapeutic from older users’ own mobile phones, this study had a high attrition rate, similar to other recent studies [47-49] that also raised concerns about internet-based and mobile-based self-help CBT interventions. As one possible explanation for the high attrition rate, the MIND MORE app was designed as a multimodal, but more psychoeducation-focused, CBT-I app with less interactive, more informative educational content to provide app users with credible health information sourced from health professionals. For this reason, users might be less likely to continue using the main content of the sleep hygiene education after the completion of all 3 sessions, except when reviewing clipped educational content, keeping the sleep diary, or completing the thought record and constructive worry worksheet on a regular basis to track their progress over time. Another possible explanation is that the high attrition rate could result from the Google operating system or Play Store feature that might suggest the uninstallation of unused apps to optimize the operation of Android phones with a relatively low specification, even if this was slightly different depending on the mobile phone and iOS version. Another possible explanation is that this high attrition rate might be the outcome of learning how to withdraw from the MIND MORE app service, as well as how to install and uninstall the app from their devices during the 1-day training program. As this study failed to reveal the cause of the high attrition rate and what will contribute to improve retention, future research needs to collect the users’ feedback as a result of their user experiences by conducting a focus group or in-depth interview after the intervention phase, thereby effectively managing expectations and enhancing treatment adherence.

### Limitations and Future Direction

As a retrospective study, this study had some limitations based on the study protocol and sample. This sleep program protocol using the MIND MORE app was not designed for the purpose of academic research, but without particular focus on examining the efficacy of the mobile app–based CBT-I for a long-term intervention period. First, the assessment of subjective sleep quality was administered at baseline (1-day training program at the SMCD) and 1 week after the 1-week home-based self-help CBT-I. Considering that the PSQI-K is an explicit measure of usual sleep habits over the last month, it is possible that the intervention period was insufficient to examine improvements in subjective sleep quality related to use of the MIND MORE app. Moreover, PSQI-K scores should be compared between two intervention groups, namely a nonclinical or subclinical group and a group of patients with insomnia, before and after use of the MIND MORE app because the intervention group (n=9) who completed the 1-week home-based self-help CBT-I consisted of only normal-risk and high-risk older adults (Table 1).

Second, particularly when using this digital therapeutic for insomnia in clinical and academic research, a digital placebo effect and the need for an appropriate control group should be considered in future studies. Accordingly, an experimental group using the MIND MORE app should be compared with an active control group using a credible sham app, not with an inactive control group in a wait-list condition, in a double-blind, randomized controlled clinical trial. Regarding concerns that this study sample was biased to the elderly, who were more interested in digital technology use itself, mobile phone–based mental health intervention, or participation in community center activities, the effectiveness of the MIND MORE app will be investigated in a randomly selected community sample of the older population. Furthermore, the number of attendants involved in the 1-week intervention was too small to conduct statistical analysis, and the ratio of men to women was imbalanced.

Finally, the study sample was derived from a population of community-dwelling older adults in a single urban area. Differences might exist between interurban areas or between urban and rural areas. For these reasons, the findings of this study cannot be generalized to community-dwelling older adults in the general and clinical population without further research.

### Conclusions

Despite this retrospective approach to data sourced from a mobile app–based CBT-I program, which was not designed for research purposes, in an urban community center for dementia, this study provides empirical evidence that the newly developed MIND MORE app was not only usable among older users but also could improve their subjective sleep quality after the 1-week self-help intervention period. Based on the findings of this study, more diverse research protocols should be designed and administered to examine the treatment effects of the mobile self-help CBT-I intervention as an alternative, first-line treatment for insomnia, thus leading to translation from research into practice.

## Appendix

Multimedia Appendix 1 English-translated version of screen shots for the MIND MORE app (Figure 1).

Multimedia Appendix 2 English-translated version of screen shots for the MIND MORE app (Figure 2).

Multimedia Appendix 3 English-translated version of screen shots for the MIND MORE app (Figure 3).

Multimedia Appendix 4 English-translated version of screen shots for the MIND MORE app (Figure 4).

Multimedia Appendix 5 English-translated version of screen shots for the MIND MORE app (Figure 5).

## Abbreviations

CBT-I: cognitive behavioral therapy for insomnia
EOL: ease of learning
EOU: ease of use
GDS-15: 15-question version of the Geriatric Depression Scale
mHealth: mobile health
PD: perceived difficulty in using the mobile app
PSQI: Pittsburgh Sleep Quality Index
PSQI-K: Korean version of PSQI
SATIS: satisfaction
SGDS-K: Korean version of the GDS-15
SMCD: Seoul Metropolitan Center for Dementia
SMCQ: Subjective Memory Complaints Questionnaire
USE: Usefulness, Satisfaction, and Ease of Use questionnaire
