# Supplementary figures and images for: Mobile App Use for Insomnia Self-Management in Urban Community-Dwelling Older Korean Adults: Retrospective Intervention Study
Source: JMIR Mhealth Uhealth. 2020 Aug 24;8(8):e17755. doi: 10.2196/17755 (PMC7477668; doi:10.2196/17755)

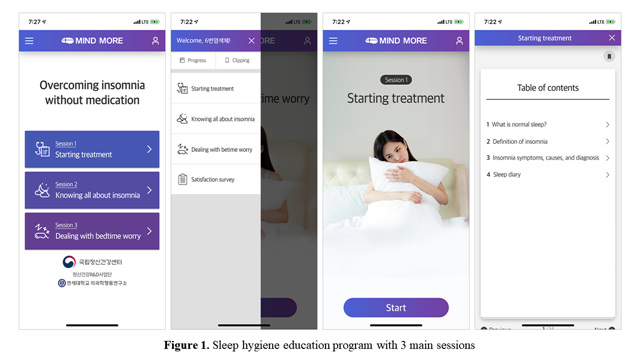

Supplement: Multimedia Appendix 1 [file mhealth_v8i8e17755_app1.png]

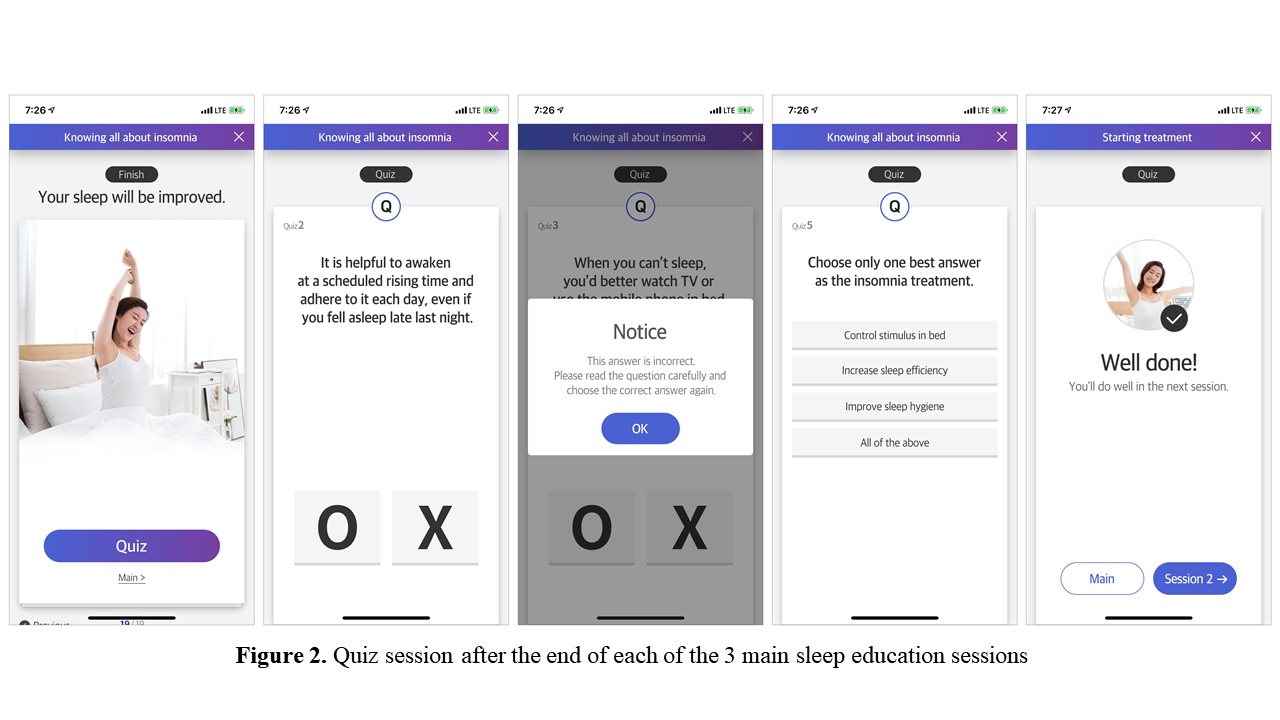

Supplement: Multimedia Appendix 2 [file mhealth_v8i8e17755_app2.png]

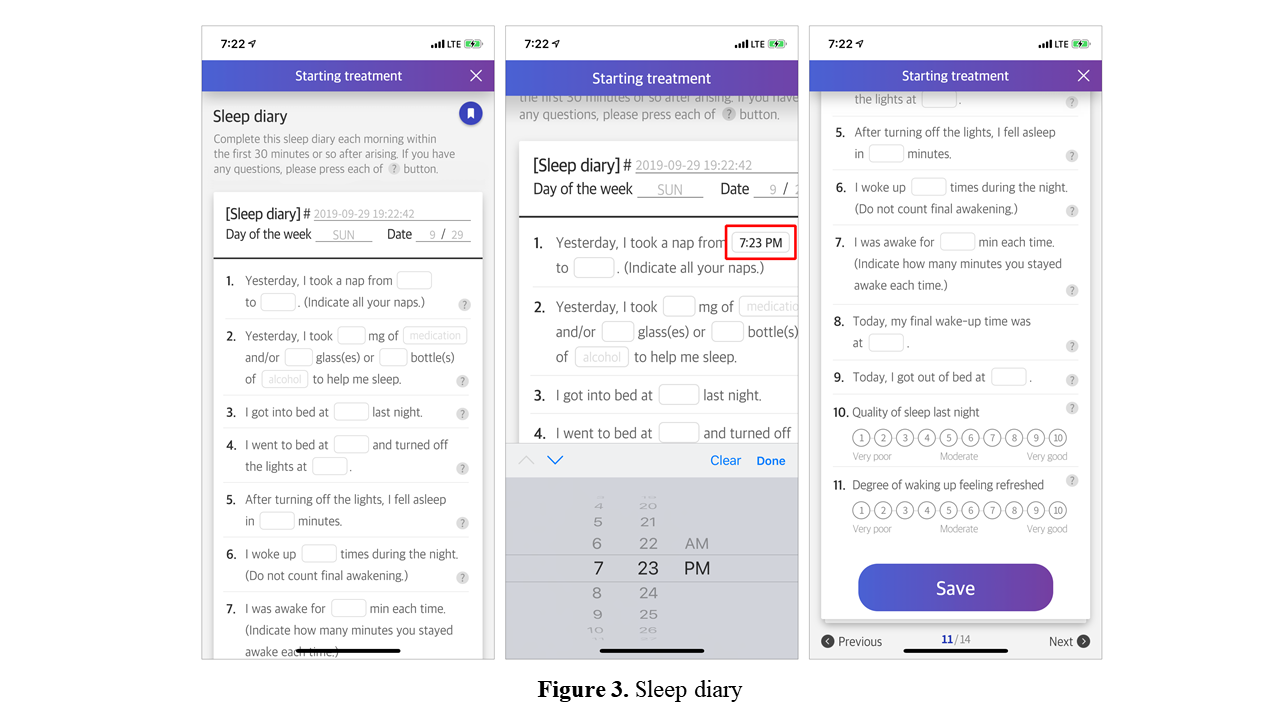

Supplement: Multimedia Appendix 3 [file mhealth_v8i8e17755_app3.png]

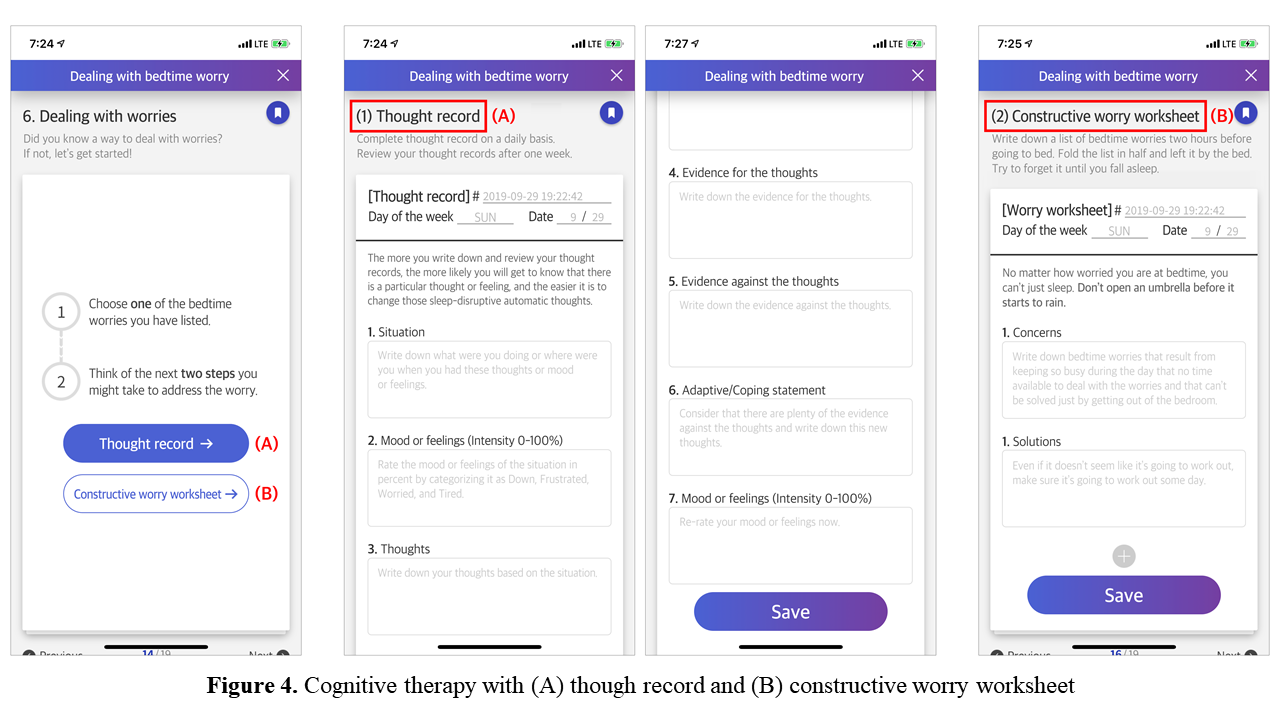

Supplement: Multimedia Appendix 4 [file mhealth_v8i8e17755_app4.png]

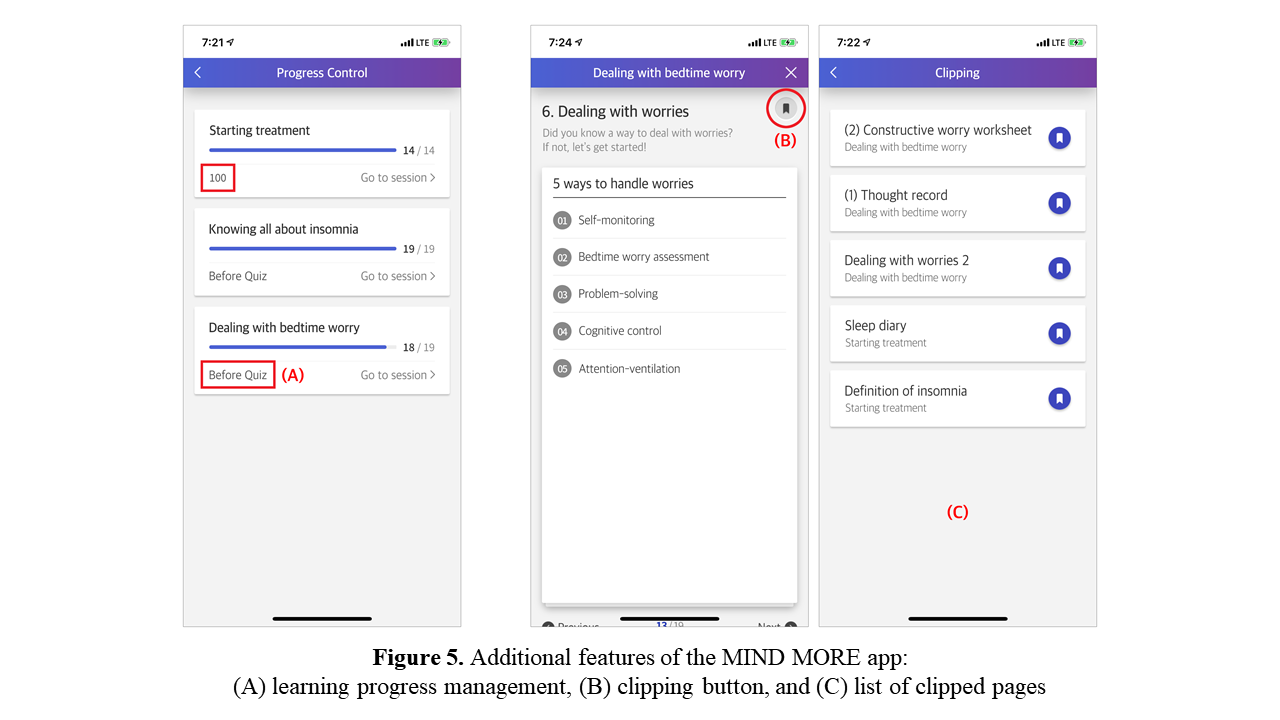

Supplement: Multimedia Appendix 5 [file mhealth_v8i8e17755_app5.png]
